# Preparation for Future Care in Community-Dwelling Older Adults With Prefrailty and Frailty: Development and Pilot Test of a Digital Intervention

**DOI:** 10.2196/92492

**Published:** 2026-08-05

**Authors:** Qiqi Ni, Jing Dong, Weilin Jiang, Jia Yi, Ran Yan, Yuhan Chen, Li Wang, Yiyu Zhuang

**Affiliations:** 1 Nursing Department, Sir Run Run Shaw Hospital, No. 3, Qingchun East Road, Shangcheng District, Hangzhou, Zhejiang, 310000, China, 86 13588708076

**Keywords:** preparation for future care, community-dwelling, digital intervention, frailty, pilot test

## Abstract

**Background:**

China’s rapidly aging population and high burden of frailty make proactive preparation for future care increasingly urgent, yet few older adults engage in such preparation. Existing interventions often overlook the heterogeneity between prefrail and frail populations and lack precision-oriented digital strategies.

**Objective:**

Building on a series of prior empirical studies, this study aimed to develop and pilot-test a digital intervention to support preparation for future care among community-dwelling older adults with prefrailty and frailty.

**Methods:**

The “Yi Yang Plan,” a WeChat mini-program, was developed through a rigorous multiphase process, including a scoping review, a convergent mixed methods study, and a structural equation model. These findings informed the design of a tailored, stage-specific intervention targeting the distinct needs of prefrail and frail older adults. An interdisciplinary team subsequently refined the intervention using an iterative design approach. The intervention modules comprised 4 processes: Awareness Enhancement, Care Resources, Care Decision-Making, and Care Planning. Pilot testing involved expert panel consultations and think-aloud tests. Expert characteristics, engagement, and authority coefficients were assessed, and feedback was synthesized using content analysis. Feasibility and acceptability among older adults and their family members were evaluated through task completion metrics, satisfaction surveys, think-aloud protocols, and semistructured interviews.

**Results:**

Seven experts participated in the consultation, with an engagement coefficient of 100% and an authority coefficient of 0.88. Recommendations were synthesized into four key themes: (1) establishing mechanisms for care plan updating and review, (2) strengthening user-centered design, (3) implementing dynamic resource management and robust data security measures, and (4) enhancing integration with community care systems and policy frameworks. A total of 20 older adults and 20 family members were recruited. All participants successfully completed the assigned tasks, with a mean completion time of 20.7 (SD 5.2) minutes. Satisfaction ratings were generally favorable. The qualitative findings indicated that the intervention was perceived as useful and professionally designed, while also identifying several challenges, including variability in preparation for future care readiness and educational levels, limited interactivity, insufficient practical content, and reliance on support from adult children. Suggested improvements included enhanced personalization, additional supportive tools, improved usability, greater involvement of adult children, and stronger integration with offline services.

**Conclusions:**

The “Yi Yang Plan” demonstrated preliminary scientific validity, feasibility, and acceptability as a multidisciplinary, collaboratively developed digital intervention to support preparation for future care among older adults with prefrailty and frailty. By translating prior empirical and theoretical findings into a differentiated, precision-oriented digital strategy, this study advances interventions beyond conventional one-size-fits-all approaches. Future iterations should focus on optimizing system architecture, user interface design, platform functionality, and implementation strategies. Further research should conduct higher-quality randomized controlled trials to evaluate the platform’s effectiveness.

## Introduction

In China,[1] older adults are defined as those aged 60 years and above [2]. By the end of 2024, the population of older adults in China reached approximately 310 million, accounting for 22% of the total population [3]. By 2035, older adults are expected to account for more than 30% of the population [4]. With advancing age, physiological reserve capacity progressively declines or becomes dysregulated, leading to increased vulnerability, reduced resilience to stressors, and impaired maintenance of internal homeostasis; consequently, older adults are more likely to develop frailty [5,6]. Frailty is a complex geriatric syndrome characterized by multifactorial etiologies and diverse influencing factors. Its hallmark features include reduced muscle strength, decreased endurance, and progressive physiological functional decline, which collectively increase the risks of dependency and mortality [7,8]. Evidence indicates that frailty may be associated with a 15% to 50% increase in all-cause mortality risk [9] and that for every 0.1-unit increase in the frailty index, the risk of adverse health outcomes rises by 68% [10]. Epidemiological studies suggest that the prevalence of frailty is approximately 15% among adults aged 65 years and older, increasing to 25% to 50% among those aged 80 years and above [11-13]. A systematic review further reported that the prevalence of frailty among community-dwelling older adults in China ranges from 5.9% to 17.4%, with a pooled prevalence of 10.0% [14]. The progression of frailty leads to a gradual decline in older adults’ ability to live independently and may ultimately result in disability, necessitating reliance on external care and imposing substantial caregiving burdens on families and society [15]. Projections indicate that by 2050, approximately 91.4 million older adults in China will require long-term care services [16], reflecting a growing and sustained demand for formal care provision.

Under the influence of the cultural norm of filial piety, the traditional belief of “raising children for old-age support,” and legal as well as moral regulations mandating adult children’s caregiving responsibilities, older adults in China have traditionally relied predominantly on family-based care models [17,18]. However, as a result of the long-standing one-child policy, family structures have undergone profound changes, with nearly 70% of households nationwide becoming single-child families [19]. At present, the parents of the first generation of only children have entered old age, and over the next 3 decades, the number of older and oldest-old parents with a single child is expected to continue to increase. China is therefore approaching a peak period of “only-child-supported aging” [20]. Nevertheless, only children face substantial practical challenges in fulfilling eldercare responsibilities. Due to work-related pressures and obligations related to raising the next generation, they often struggle to provide adequate financial support, daily care, and emotional companionship for their aging parents [21]. In addition, with accelerating modernization and urbanization, increasing female labor force participation, and rising life expectancy among older adults, the traditional family caregiving function has been further weakened. Against this backdrop, older adults are increasingly compelled to shift from traditional family-based care models to transitional or more formalized, socially provided care arrangements [22]. This transition necessitates that older adults proactively plan for future care to mitigate potential caregiving risks. Taken together, in the face of the complex care needs associated with frailty, the traditional family care model is no longer sufficient to meet current demands. There is an urgent need for frail older adults to play a more active role by engaging in early planning, mobilizing available resources, and preparing for future care.

Preparation for future care (PFC) refers to the process of preparing for potential needs for assistance with activities of daily living resulting from functional decline due to chronic illness or health-related events in the future [23]. A substantial body of literature indicates that PFC promotes active aging [24]. Older adults who engage in more systematic planning for future care report a greater sense of control over their lives [25], higher life satisfaction [26], and lower levels of anxiety and depression [27]. Moreover, as a form of long-term risk management, PFC enables advance arrangements for future care needs, such as identifying potential caregivers, selecting care institutions, or planning for transitions in care modalities, which can substantially alleviate caregiver burden and generate benefits for caregivers as well [24]. In contrast, a lack of PFC is associated with higher risks of illness and increased financial and emotional strain [28]. Therefore, actively guiding older adults to engage in PFC is a critical component of community nursing practice in the context of population aging, with significant practical relevance and social value.

Over the past 2 decades, most interventions have focused on short-term care planning for patients at the end of life, with limited attention given to long-term care preparation among older adults [29,30]. Existing PFC interventions are predominantly delivered through face-to-face communication or group meetings and rely largely on experiential guidance, often lacking robust empirical evidence and theoretical frameworks. In addition, many studies are characterized by methodological limitations and poor scalability and feasibility for widespread implementation [31]. Consequently, developing scientifically grounded, feasible, and easily disseminated strategies to promote PFC has become a key challenge in this field. With the rapid advancement of information technology, digital nursing models have emerged as an important area of research. For example, Hoffman et al [32] developed a web-based platform that provides education, values clarification, resources, and behavioral planning for long-term care, which effectively facilitated PFC engagement among older adults and has been adopted by health care organizations. However, most existing digital PFC interventions have been developed in Western countries, particularly the United States [33]. These platforms often lack health care professional–led guidance and are not grounded in explicit theoretical frameworks, limiting their capacity to address the individualized needs of frail older adults [34].

In recent years, the Chinese government has issued a series of policy documents [35-37] emphasizing the development of nursing information networks and service platforms to deliver efficient and accessible digital nursing services to older adults in need. In light of both practical demands and policy priorities, there is an urgent need to develop a theory- and evidence-informed digital PFC intervention platform tailored to the Chinese context. Such a platform could enhance frail older adults’ awareness of the importance of PFC and facilitate the implementation of practical long-term care plans, thereby optimizing the prospective allocation of aging-related resources. Therefore, the objectives of this study are twofold: (1) to develop a localized and stratified digital intervention for supporting PFC among community-dwelling older adults with prefrailty and frailty and (2) to assess the scientific validity, feasibility, and acceptability of this digital intervention using expert panel consultations and think-aloud tests. These efforts will provide a foundation for future refinements of the platform and a subsequent randomized controlled trial.

## Methods

### Digital Program Development: Research Foundation

**Figure 1.**
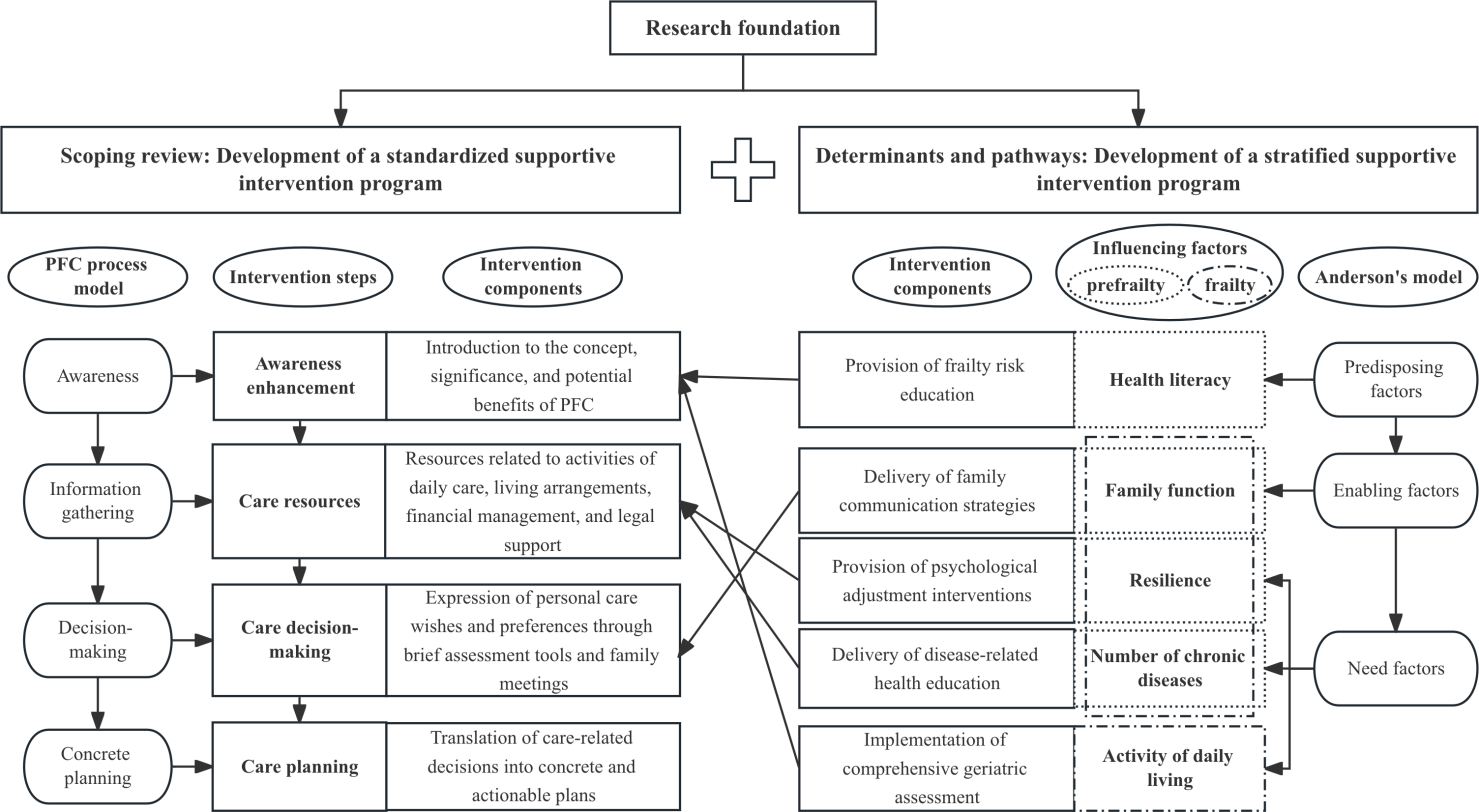
Research foundation of digital intervention development. PFC: preparation for future care.

To inform the development of a digital intervention for PFC among community-dwelling older adults with frailty, we conducted a series of sequential studies, including a scoping review [33], a convergent mixed methods study [38], and a structural equation model (SEM) based on triangulated findings from the mixed methods study. In brief, the scoping review identified 4 core components of PFC-supportive interventions (Awareness Enhancement, Care Resources, Care Decision-Making, and Care Planning), which are consistent with the PFC process model proposed by Sörensen et al [39]. The mixed methods study, guided by the Andersen Behavioral Model of Health Services Use, identified key determinants of PFC among prefrail and frail older adults. Furthermore, the SEM revealed that influencing pathways of PFC differ between prefrail and frail older adults, providing empirical support for a stratified intervention design (Figure 1).

**Figure 2.**
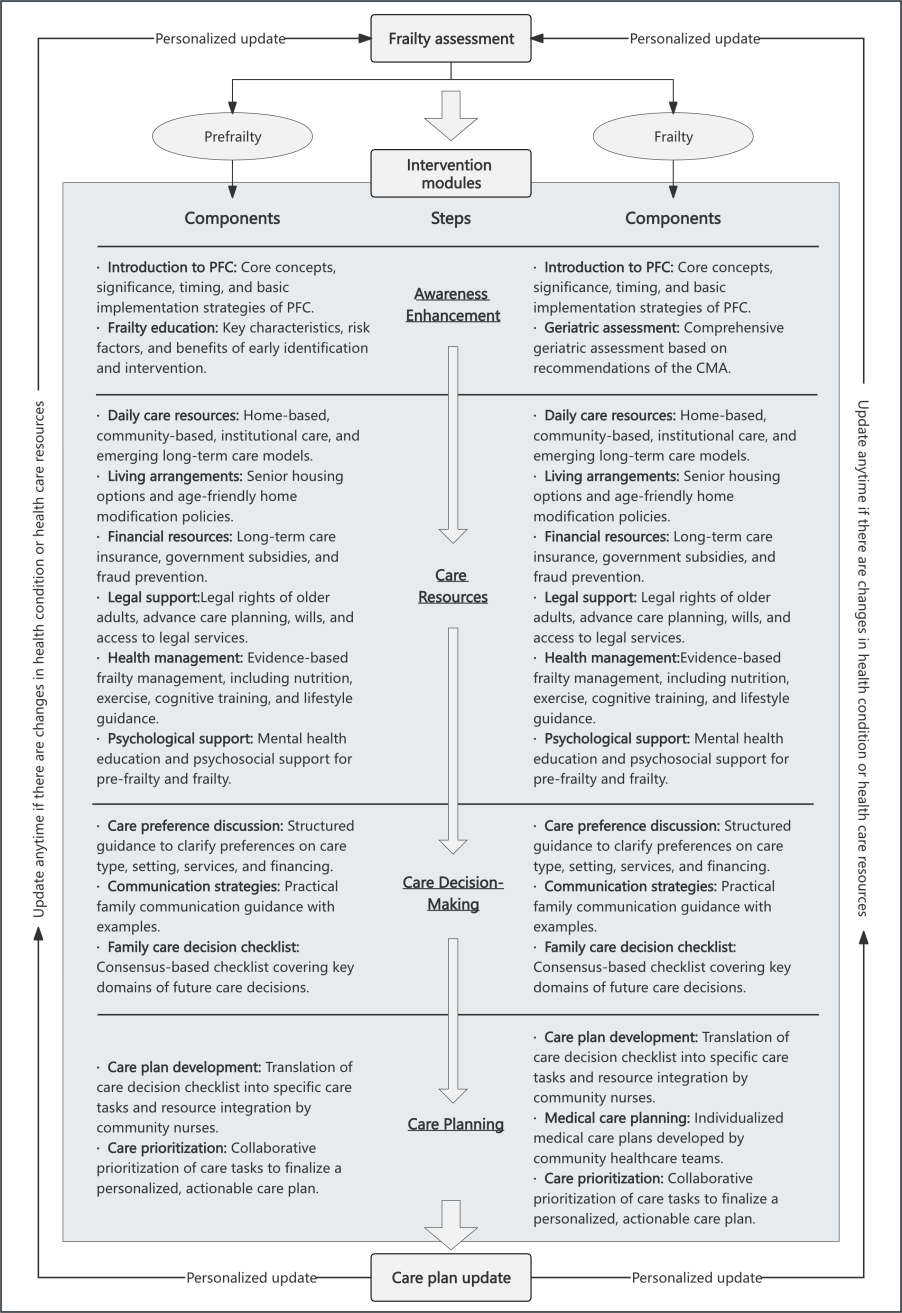
Supportive intervention program for preparation for future care among community-dwelling prefrail and frail older adults. CMA: Chinese Medical Association; PFC: preparation for future care.

Based on these findings, a supportive intervention program for PFC among community-dwelling frail older adults was developed (Figure 2). The program emphasizes tailoring intervention strategies according to frailty status to enable targeted and differentiated support. Specifically, for prefrail older adults, interventions focus on frailty education, health management, psychological support, and family communication. For frail older adults, interventions focus on comprehensive geriatric assessment, health management, psychological support, and medical care planning.

### Digital Program Construction

#### Platform Selection and Conceptualization

Based on the intervention program, the digital platform was developed as a WeChat mini-program, given that WeChat is the most widely used mobile app in China and offers high accessibility for older adults. The mini-program was named “Yi Yang Plan.” In Chinese, “Yi Yang” refers to maintaining physical and mental well-being through appropriate care, reflecting the program’s goal of promoting healthy and meaningful aging.

#### Research Team Formation

An interdisciplinary team was established, comprising 2 geriatric care experts with experience in digital health app design, 1 interface designer specializing in age-friendly design, 1 software engineer, and 1 doctoral student in nursing. The team collaboratively conducted the design, development, and refinement of the mini-program.

#### Interface Design and Development Process

The platform interface was designed using an iterative approach. Initially, the interface designer created preliminary prototypes based on the intervention protocol. These prototypes were reviewed by the research team on a page-by-page basis, followed by iterative revisions focusing on content clarity, navigation logic, and user experience. After multiple rounds of revisions and optimization, the finalized design was implemented by the software engineer. The interface design was developed using the Lanhu platform (version 6.0.4; Beijing Jinwei Zhiguang Information Technology Co; Multimedia Appendix 1).

#### Development Environment

The mini-program was developed within the official WeChat Mini Program development environment. A developer account was registered, and a unique application identification was obtained. The WeChat Developer Tools were used to support coding, debugging, and deployment, with access to standardized frameworks, components, and technical documentation. Upon completion, the software engineer uploaded and managed the program code.

#### System Architecture

**Figure 3.**
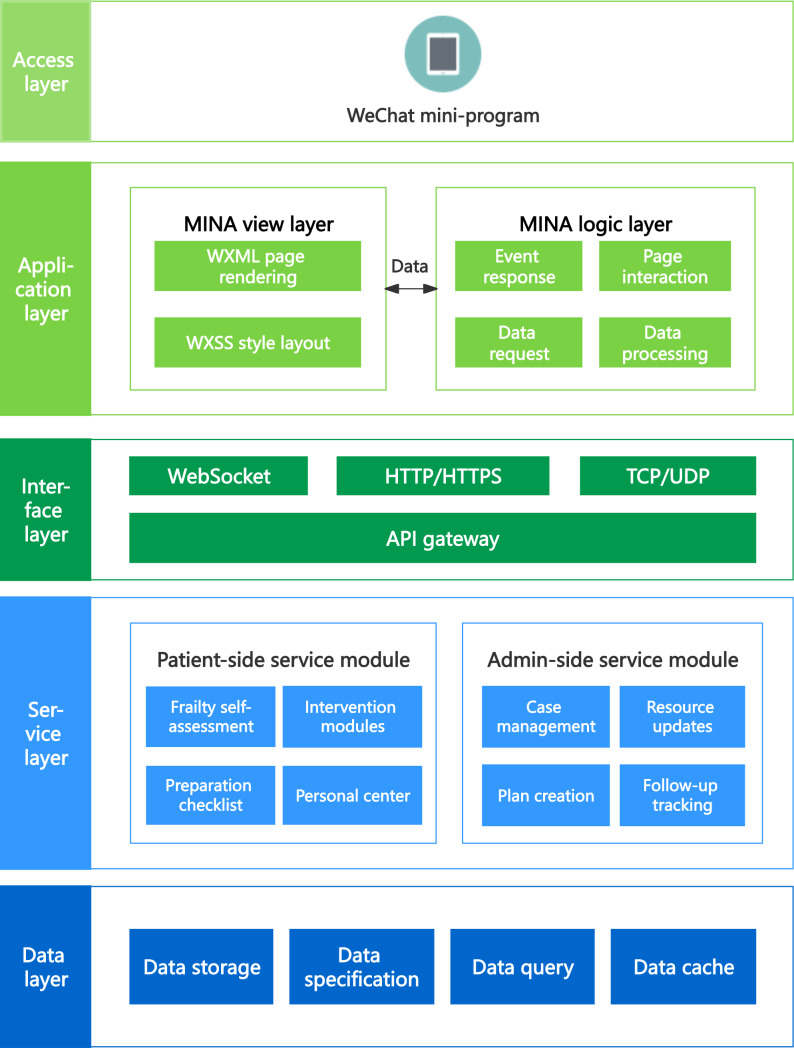
System architecture of the “Yi Yang Plan” mini-program. MINA: the WeChat Mini Program; TCP: Transmission Control Protocol; UDP: User Datagram Protocol; WXML: WeiXin Markup Language; WXSS: WeiXin Style Sheets.

The system adopts a front-end or back-end separated architecture. The front-end was developed using the uni-app framework with uView components, while the back-end was implemented using C#-based server-side services. Data exchange between the 2 layers is achieved through network communication protocols. The platform architecture is organized into 5 hierarchical layers: Data Layer, Service Layer, Interface Layer, Application Layer, and Access Layer. This layered architecture enhances system scalability, maintainability, and security (Figure 3).

#### Cognitive Load-Oriented Design

Complex content, such as financial and legal information, was simplified through multimodal presentation formats. Specifically, short videos were used to explain key concepts, text-to-speech functionality was incorporated for essential content, and illustrative images were added to facilitate comprehension.

### Digital Platform Functions

#### Overview

The “Yi Yang Plan” consists of 2 primary components: a community management terminal and a patient or family app terminal. The community management terminal supports case registration, list management, care plan development, resource updates, and follow-up tracking (Multimedia Appendix 2). The patient or family app terminal comprises 4 main modules: self-assessment, intervention modules, care preparation checklist, and personal center.

#### Self-Assessment

After account registration and binding (Multimedia Appendix 3A), users complete a frailty self-assessment (Multimedia Appendix 3B). Based on the results, users are classified as prefrail or frail, and a corresponding personalized intervention pathway is initiated (Multimedia Appendix 3C).

#### Intervention Modules

Guided by the PFC process model [39], the intervention is operationalized through four sequential and functionally integrated modules:

##### Awareness Enhancement

This module introduces the concept, importance, timing, and strategies of PFC through standardized short videos (Multimedia Appendix 4A). For prefrail users, targeted frailty education is provided, focusing on risk factors and early intervention (Multimedia Appendix 4B). For frail older adults, the module incorporates an electronic comprehensive geriatric assessment recommended by the Chinese Medical Association [40], including the Simplified Nutritional Appetite Questionnaire, SARC-F (S‌trength, A‌ssistance in walking, R‌ise from a chair, ‌C‌limb stairs, F‌alls), and Rapid Cognitive Screen (Multimedia Appendix 4C).

##### Care Resources

This module provides a structured repository of evidence-informed care resources (Multimedia Appendix 5A). Resources include daily care services, housing and home modification policies, financial planning, legal support, health management, and psychological support (Multimedia Appendix 5B-P). Information is delivered in multiple formats, including text, images, audio, and video.

##### Care Decision-Making

This module facilitates shared decision-making and family engagement. Structured discussion guides (Multimedia Appendix 6A) and communication strategies (Multimedia Appendix 6B) support family discussions. Users then complete a 12-domain family care decision checklist (Multimedia Appendix 7), reflecting consensus among family members (Multimedia Appendix 6C).

##### Care Planning

Based on the completed checklist, community nurses develop an initial care plan using the management terminal. For frail older adults, individualized medical care plans are additionally formulated. Users review, adjust, and prioritize care tasks within the mini-program, resulting in a finalized personalized care plan (Multimedia Appendix 8A-B). The system also supports ongoing plan updates (Multimedia Appendix 8C).

### Care Preparation Checklist

Upon the completion of the intervention modules, the system automatically generates a comprehensive care preparation checklist integrating care plans and related resources. This checklist can be exported in PDF format for family communication or offline consultations (Multimedia Appendix 9A).

### Personal Center

The Personal Center module allows users to manage their personal profiles and maintain relevant information. It also includes a “favorites” function, enabling users to save and access important resources for future reference (Multimedia Appendix 9B).

### Expert Panel Consultations

#### Participants

A purposive sampling method was used to recruit experts from 6 fields: nursing management, community nursing, geriatric nursing, general medicine, geriatric medicine, and sociology. Experts were eligible if they met the following criteria: (1) demonstrated recognized academic expertise and authority in the treatment, care, or long-term care of community-dwelling frail older adults; (2) had experience in leading or participating in digital health-related projects; (3) held an associate senior professional title or higher; and (4) had at least 10 years of relevant professional experience.

#### Procedure

Prior to the consultation, all experts received the study protocol, a trial access link to the mini-program, and the meeting agenda. The consultation was conducted in a hospital conference room. At the beginning of the session, the project leader introduced the study background and objectives. With participants’ informed consent, the entire session was audio-recorded. Experts were then guided through a 20-minute hands-on use of the “Yi Yang Plan” mini-program. The consultation focused on 3 key domains: scientific validity, operability, and feasibility of the intervention. During the discussion, the facilitator ensured that all questions were clearly understood and that ambiguities were resolved to accurately capture expert perspectives. Within 24 hours, audio recordings were transcribed verbatim and independently verified by 2 researchers. The transcripts were subsequently returned to the experts for member checking to confirm the accuracy and completeness of their statements.

#### Data Collection

##### Expert Characteristics

Data included gender, age, highest degree, professional title, field of expertise, and years of experience.

##### Expert Engagement Coefficient

Expert engagement was assessed using the response rate, reflecting the level of participation and attentiveness of experts in the consultation process. A higher response rate indicates greater engagement and contributes to the objectivity of the consultation results.

##### Expert Authority Coefficient

The authority coefficient was used to evaluate the credibility and expertise of the participating experts. It reflects their professional judgment and familiarity with the topic. An authority coefficient of 0.70 or above is generally considered indicative of reliable expert consultation. Detailed calculation methods are provided in Multimedia Appendix 10.

### Data Analysis

Descriptive analysis was conducted to summarize expert demographics, engagement coefficients, and authority coefficients. Qualitative data from the consultation were analyzed using conventional content analysis. Transcripts were independently coded by 2 coders, followed by face-to-face discussions to modify and reconcile coding discrepancies. Data analysis was performed using MAXQDA 2024 (VERBI Software).

### Think-Aloud Test

#### Participants

Using convenience sampling, we recruited 20 dyads consisting of community-dwelling older adults with prefrailty and frailty and their family members from a community health center in Hangzhou, China. According to Faulkner [41], 20 participants are sufficient to identify the majority of usability issues. The inclusion criteria for older adult participants were as follows: (1) aged 60 or above years; (2) self-reported as prefrail or frail based on the FRAIL (F‌atigue, R‌esistance, A‌mbulation, ‌I‌llness, L‌oss of weight) scale (1‐2 criteria indicating prefrail; ≥3 criteria indicating frail); (3) residence in the community for at least 6 months [42]; (4) ability to read, write, and understand Mandarin; and (5) ability to use a smartphone. Individuals with a clinical diagnosis history of severe psychiatric or neurological disease, such as schizophrenia or Alzheimer disease, were excluded. Each older adult was encouraged to involve 1 family member (aged ≥18 years), who was related to the patient by blood, marriage, adoption, or affinity as a significant other, and met the language and smartphone-use criteria.

#### Procedure

Think-aloud tests were conducted in a meeting room arranged to resemble a typical living room environment. Before each session, we prepared the necessary equipment: smartphones with the WeChat preinstalled for the participants, audio recorders, timers, and R‌esistance and pens for observers. We also enabled screen-recording software on the smartphones to capture on-screen interactions. Based on the core functions provided by the “Yi Yang Plan” mini-program and the typical operations required for preparing for future care, 5 core tasks were designed in consultation with a software engineering expert (Multimedia Appendix 11).

At the beginning of each session, researchers introduced the study’s purpose. Participants were informed that their verbalizations and screen operations would be recorded during the testing process, and their data were fully confidential. Participants were also informed about the think-aloud protocol and instructed to verbalize their thoughts continuously while using the mini-program. As they navigated through each feature of the mini-program, they were encouraged to speak aloud their current thoughts; any decisions they were considering; and any feelings, confusions, or difficulties they experienced. Importantly, researchers did not intervene or answer questions during the task; participants were advised beforehand that any questions or issues they raised would be addressed only after the completion of the session. If a participant became stuck or hesitant, researchers offered neutral encouragement to continue but provided no direct assistance. If certain tasks could not be completed by the participant, these were noted as uncompleted tasks in our observations.

Each test session was recorded in multiple ways. A digital audio recorder captured all spoken comments from participants, the smartphone’s screen-recording software logged the exact on-screen actions and navigation, and the timer recorded the duration of each completed task. Researchers also objectively observed and documented each participant’s spoken words and nonverbal behaviors without attempting to interpret them.

#### Measures

##### General Characteristics

For older adults, the data included age, gender, residence, education level, and prior experience with mobile health apps. For family members, the data included relationship to the older adults, age, education level, and prior experience with mobile health apps.

##### Platform Use Satisfaction

Platform use satisfaction was assessed using a self-developed questionnaire comprising 6 items: interface design, features and functions, ease of operation, content quality, system stability, and overall experience. Each item was rated on a 5-point Likert scale, with scores ranging from 1 (“very dissatisfied”) to 5 (“very satisfied”).

##### Platform User Experience Interviews

A semistructured interview guide was developed based on the technology acceptance model (TAM) [43], including three open-ended questions: (1) Do you perceive this platform as useful? (2) Do you find this platform easy to use? (3) What suggestions do you have for improving the platform?

### Data Analysis

Quantitative data, including task completion time and satisfaction scores, were analyzed using descriptive statistics. Categorical variables were summarized as frequencies and percentages, and continuous variables were reported as means (SD). The qualitative data from think-aloud sessions and interviews were transcribed verbatim and analyzed within the framework of the TAM. Thematic analysis and coding were conducted with a focus on the core dimensions of perceived usefulness and perceived ease of use in order to elucidate participants’ acceptance motivations, perceived barriers, and suggestions related to their use of the “Yi Yang Plan” mini-program. Data analysis was performed using MAXQDA 2024 (VERBI Software). Data analysis was conducted in Chinese. Themes and related quotations were translated into English for report writing by the first author. Translations were reviewed by the research team, and discrepancies were resolved through discussion.

### Ethical Considerations

This study followed the principles of the Declaration of Helsinki and was approved by the Ethics Research Committee of Sir Run Run Shaw Hospital Affiliated to Medical College of Zhejiang University (2024-2759-01). Before enrollment, the responsible researcher explained the possible benefits and risks of the study to each potential participant in a face-to-face conversation, and they could withdraw from the study at any time without penalty. Sufficient time was provided for individuals to consider their participation, and only those who voluntarily agreed and signed the informed consent form were included.

## Results

### Expert Panel Consultations

#### Expert Characteristics

A total of 7 experts were recruited, including 6 female experts and 1 male expert. The mean age was 52.9 SD (7.38) years, and the average year of professional experience was 30.4 (SD 10.62) years. Detailed demographic characteristics are presented in Table 1.

**Table 1.** Demographics characteristics of the experts (n=7).

No.	Age (y)	Gender	Highest degree	Professional title	Field of expertise	Years of experience
1	50	Female	Master	Chief physician	General medicine	27
2	57	Female	Master	Chief physician	Geriatrics	34
3	60	Female	Master	Professor	Geriatric nursing	41
4	45	Female	Master	Chief physician	General medicine	22
5	40	Male	PhD	Professor	Sociology	10
6	58	Female	Master	Associate chief nurse	Community nursing	39
7	60	Female	Master	Professor	Nursing management	40

#### Expert Engagement Coefficient

All experts actively participated in the consultation and provided constructive feedback on the digital intervention. The engagement coefficient was 100%, indicating full participation and a high level of involvement.

#### Expert Authority Coefficient

The calculated authority coefficient was 0.88, indicating a high level of expertise and credibility among the participating experts. Therefore, the consultation results can be considered reliable.

#### Expert Recommendations

##### Establish a Mechanism for Care Plan Updates and Reviews

Experts emphasized that future care planning is a dynamic and evolving process. Expert 3 emphasized that care plans should be adjusted according to changes in older adults’ physical conditions, family structures, personal beliefs, and policy directions. Accordingly, a regular update mechanism is necessary, with experts recommending updates every 6 months. This frequency balances the need for timely adaptation without overburdening older adults or their families. Experts 6 and 7 agreed with this recommendation. Additionally, expert 1 suggested incorporating a feature that allows users to review past care plans, enabling older adults and their families to compare changes over time and understand the rationale behind adjustments. Therefore, the platform should implement a comprehensive mechanism for both updating and reviewing care plans to enhance their timeliness, continuity, and adaptability.

##### Strengthen User-Centered Design

Several experts highlighted the lack of user-centered design in the current platform. Expert 2 strongly recommended that the platform architecture should address the real needs of older adults, enabling users to quickly access personalized and valuable information upon opening the app. Expert 5 noted that the platform’s text-heavy content is not suitable for older adults with limited digital literacy. Excessive text undermines usability, while a lack of personalized content reduces usefulness. This expert suggested using more videos, intuitive interactions, and case stories to reduce cognitive load. Expert 4, from a community practice perspective, emphasized that the platform must address practical problems faced by older adults, reinforcing the necessity of a needs-driven approach. Therefore, user experience design must go beyond functionality, aiming to create a digital environment that is friendly, inclusive, and capable of effectively conveying complex information to older adults.

##### Implement Dynamic Resource Management and Data Security Measures

The platform’s long-term viability depends on content accuracy and technological advancement. Experts expressed concerns about static resources and limited technical support. Expert 2 highlighted that outdated resources compromise credibility and usefulness, prompting users to seek alternative information sources. Expert 7 underscored the importance of data security, continuous technical support, and ongoing updates, noting that nursing researchers may lack the technical expertise required, necessitating interdisciplinary collaboration. This suggests that a platform lacking dynamic maintenance and professional technical support would have limited real-world effectiveness. Therefore, the platform should ensure dynamic content updates and implement strict data security measures.

##### Enhancing Integration With Community Care Systems and Policy Frameworks

The ultimate value of the platform lies in its ability to address real-world needs. Expert 6, a community practitioner, emphasized that the platform’s value increases if it can provide actionable guidance and integrate with offline services. Expert 1 highlighted the importance of aligning the platform with national public health policies. This indicates that the platform should not function as an isolated information source; rather, it should be embedded within the existing care ecosystem, serving as a bridge between user needs and professional services. Active integration with public service systems, enabling data sharing and online appointment scheduling, would enhance accessibility and efficiency. Additionally, collaboration with community organizations and the selection of representative communities for pilot studies are recommended to evaluate effectiveness in practice.

### Think-Aloud Test

#### Participant Characteristics

A total of 20 dyads were recruited, including 20 community-dwelling older adults and 20 family members. Among the older adults, 15 (75%) were classified as prefrail, and 13 (65%) were male participants. The mean age was 72.2 (SD 8.65) years, with 9 (45%) participants aged between 70 and 79 years. Regarding educational attainment, 8 (40%) participants had completed junior high school. Thirteen (65%) participants reported prior experience using mobile health apps. Among family members, 8 (40%) were daughters of the older adults. The mean age was 51.9 (SD 13.04) years, with 7 (35%) participants aged between 40 and 49 years. Eleven (55%) had a university-level education or higher, and 17 (85%) had prior experience using mobile health apps (Table 2).

**Table 2.** Characteristics of the participants (n=40).

Characteristics	Values
Older adults (n=20)	
Frailty status, n (%)	
Prefrailty	15 (75)
Frailty	5 (25)
Gender, n (%)	
Male	13 (65)
Female	7 (35)
Age (y),	
Mean (SD)	72.2 (8.65)
60‐69, n (%)	8 (40)
70‐79, n (%)	9 (45)
80‐89, n (%)	2 (10)
≥90, n (%)	1 (5)
Education level, n (%)	
Primary school	3 (15)
Junior high school	8 (40)
High school	5 (25)
University and above	2 (10)
Have you used a mobile health care application before?, n (%)	
Yes	13 (65)
No	7 (35)
Family members (n=20)	
Kinship with patient, n (%)	
Spouse	5 (25)
Son	7 (35)
Daughter	8 (40)
Age (y)	
Mean (SD)	51.9 (13.04)
30‐39, n (%)	4 (20)
40‐49, n (%)	7 (35)
50‐59, n (%)	2 (10)
60‐69, n (%)	4 (20)
≥70, n (%)	3 (15)
Education level, n (%)	
Primary school	3 (15)
Junior high school	2 (10)
High school	4 (20)
University and above	11 (55)
Have you used a mobile health care application before?, n (%)	
Yes	17 (85)
No	3 (15)

#### Duration of Task Test and Satisfaction Ratings

All the set tasks were completed by the older adults and their family members. The mean total time required to complete all tasks was 20.7 (SD 5.24) minutes. Among the individual tasks, participants spent the least time on system familiarization (task 1; mean 0.8, SD 0.48 min) and the most time on exploring care resources (task 3; mean 6.9, SD 1.81 min; Table 3).

User satisfaction ratings were generally favorable across system attributes. The highest rating was observed for ease of operation, with a rate of 4.1 (SD 0.69), while content quality received the lowest rating, with a rate of 3.7 (SD 0.81), and overall experience was rated at a satisfactory level, with a rate of 3.7 (SD 0.73; Table 3).

**Table 3.** The results of the duration of task test and the satisfaction ratings.

Item	Values, mean (SD)
Duration of task test (min)	
Task 1	0.8 (0.48)
Task 2	2.9 (0.95)
Task 3	6.9 (1.81)
Task 4	6.0 (1.31)
Task 5	4.0 (1.55)
Overall	20.7 (5.24)
Satisfaction ratings	
Page design	3.8 (0.89)
Features and functions	3.8 (0.70)
Ease of operation	4.1 (0.69)
Content quality	3.7 (0.81)
System stability	3.9 (0.72)
Overall experience	3.7 (0.73)

#### Views on the “Yi Yang Plan” Mini-Program

##### Perceived Usefulness of the Mini-Program

Participants expressed mixed views about the acceptability of the “Yi Yang Plan” mini-program. Some users acknowledged the program’s value. Participants noted that those with greater awareness of care planning found the content engaging and described the mini-program as professionally designed and useful. One older adult with a caregiving background strongly emphasized that such proactive planning tools are highly meaningful and necessary, highlighting that everyone will ultimately face care-related challenges. The participant stressed that the tool can help older adults recognize the importance of PFC and take preparatory actions. Some participants reported that the mini-program provided access to information and resources that had previously been difficult to obtain, which they found highly beneficial. In addition, several users also underscored that the mini-program was developed by a multidisciplinary professional team, which reinforced the credibility of its design and content:

*As aging becomes increasingly severe ... the situation of elderly care is becoming more severe … this (mini-program) is a good tool to participate in this elderly care industry earlier.*

[Male, 78 years old, high school graduate]

*This mini-program provides valuable information … I think that’s really good. For example, policies on age-friendly home modifications are very helpful for older adults. The way houses were renovated in the past might not suit them anymore.*

[Son, 48 years old, university graduate]

*I think this mini-program is very good … since it was developed by nursing professionals, and it is highly necessary.*

[Female, 76 years old, high school graduate]

Nevertheless, some users adopted a cautious or reserved stance, perceiving the direct benefits of the program for older adults as limited. Certain participants demonstrated insufficient awareness of PFC and lacked the inclination to engage in proactive planning, leading them to view the mini-program as unnecessary. In contrast, others felt that they had already completed most of their preparations and therefore regarded the program as offering little additional support. This perspective underscores that older adults occupy different stages of PFC, which shapes their varying needs for the program. In addition, some pointed out that older adults with lower levels of education were unable to understand or process the information presented in the program, leaving them without the ability to use it effectively or appreciate its value:

*A lot of older adults in rural areas can’t accept the idea of actively preparing for future care … so it’s pretty hard to promote and use this kind of mini-program.*

[Daughter, 39 years old, high school graduate]

*I’ve been planning for a long time. I already have information about a lot of nursing homes saved on my phone, and I’ve even called them to learn more.*

[Female, 62 years old, university graduate]

*I think there is some usefulness … (but) for older adults with low education levels, its role may not be very significant.*

[Male, 63 y old, university graduate]

##### Perceived Usability of the Mini-Program

Many participants emphasized that the program demonstrated a high level of usability, aligning with older adults’ operational habits and cognitive abilities, with logically structured, easy-to-operate, and comprehensive content. By providing a clear and accessible design, the mini-program was also perceived as enhancing older adults’ confidence in using digital technologies:

*It’s just a tree structure (pointing at the screen), the operation is easy too, and it feels quite straightforward. Maybe younger people think it’s not fancy enough, but for us, it’s very clear.*

[Male, 76 years old, middle school graduate]

*The pages are very clear, and the content is quite comprehensive. For older adults like me, even if we’re not super skilled with smartphones, we can still use them (with confident nodding).*

[Female, 76 years old, high school graduate]

However, many older adults reported difficulties in operating and understanding complex information due to declining vision, limited education, and insufficient digital skills. For example, some noted operational barriers within the mini-program, such as unclear return or next-page buttons that required additional effort to navigate. Others highlighted barriers in page presentation, including excessively small font sizes and overly dense text, which made reading and information acquisition challenging. In addition, participants pointed out that disease-related knowledge was described as overly specialized, with numerous technical terms that hindered comprehension. Similarly, while resources such as government documents and updated laws and regulations were viewed as authoritative, they were considered too theoretical and lacking in practical applicability:

*The return in this mini-program is a bit troublesome.*

[Male, 63 years old, university graduate]

*The words are so small. Even with glasses, I cannot see them.*

[Female, 62 years old, university graduate]

*You should explain things in simple terms, and be more concrete. Older people do not think as actively as younger ones.*

[Male, 78 years old, high school graduate]

In addition, older adults indicated that when using the “Yi Yang Plan” mini-program, they preferred the participation of their adult children. This was because they were not sufficiently skilled at operating health-related digital tools, whereas younger family members were more adept at using them, leading older adults to rely on their support. In addition, some participants pointed out that PFC is a complex intergenerational family issue that requires the joint involvement of adult children in decision-making, ensuring that older adults’ preferences are considered while also securing the support of their children:

*Many older adults with low education level cannot use the mini-program and understand it. It must be the children or younger siblings who are able to process this information.*

[Female, 62 years old, university graduate]

*For people around my age, born in the 70 s, we can think about things like preparing for future care on our own. But for my dad’s generation, it’s much harder. Most of them rely on children (shake the head helplessly).*

[Son, 48 years old, university graduate]

##### Suggestions for Improving the Mini-Program

###### Providing Personalized Recommendations

As highlighted in the preparation for future care process model proposed by Sörensen et al [39], older adults may be at different stages of preparation. Therefore, individualized planning processes and components should be recommended based on their current level of preparation. Although the mini-program includes an assessment function, it does not yet generate personalized resources or stage-specific recommendations from the assessment results. Hence, strengthening the function of personalized recommendations is necessary:

*I’ve already thought it through, so I can make decisions directly without going through all the earlier parts. ... These resources are more like a way to fill in the gaps. I just look at what I need, and I can skip whatever I don’t.*

[Female, 76 years old, high school graduate]

###### Enhancing Supportive Tools or Assistance

Some participants suggested that many older adults with lower levels of education or limited smartphone skills showed low acceptance of using the mini-program independently. To address this, they recommended providing additional supportive measures, such as organizing offline community-based training sessions or distributing instructional videos and user manuals. These forms of assistance could help older adults better understand care preparation and use the mini-program more smoothly:

*I think you need to organize in-person classes for those older adults with low education levels … They can’t understand it just by looking (at the mini-program). You’d have to explain everything to them carefully … If they could attend in person and listen, the effect would be much better.*

[Female, 62 years old, university graduate]

###### Improving Interaction and Content

Several participants emphasized the importance of simplifying navigation and enhancing content readability. They suggested establishing clearer logic for return and exit functions, as well as providing prompt guidance at key steps to minimize confusion. Participants also recommended increasing font size and adopting plain, accessible language to accommodate older users. In addition, they proposed breaking down lengthy passages into bullet points or lists and presenting information in a simplified format similar to PowerPoint slides, which they felt would improve clarity and ease of understanding:

*Reduce the amount of text, and present the main points first so that they can be quickly understood … a format similar to PowerPoint would be easier for older adults to follow. In slides, you can use visual elements, extract the key content, enlarge the font, and keep the information brief.*

[Daughter, 44 years old, university graduate, with work experience in IT]

###### Enhancing the Involvement of Adult Children

Several participants emphasized that preparation for future care is a complex issue that requires the involvement of multiple generations. Older adults often find it difficult to process the complex information contained in the mini-program and to make related decisions. As a result, they preferred that their adult children participate in the discussions and provide assistance with using the mini-program.

*It is better for older adults to use it together with their children, and the wishes of the older adults themselves are more important.*

[Male, 48 years old, university graduate]

###### Integrating Practical Service Resources

Some participants expressed a desire for the program to be linked with offline services to improve the feasibility of planning. For example, they recommended direct connections to local nursing homes, home care providers, domestic service agencies, and medical resources. Such integration was seen as a way to help older adults move from planning to action, although participants recognized that this would require cross-sector collaboration and long-term development:

*It would be better if the connection to the local community could be stronger ... Which communities have connected with certain services, such as domestic services, medical services ... If you can do a good job with this connection ... the experience would definitely be better.*

[Son, 48 years old, university graduate]

## Discussion

### Principal Findings

This study developed and pilot-tested the “Yi Yang Plan” mini-program to support PFC among community-dwelling older adults with prefrailty and frailty. The findings from expert panel consultations demonstrated the scientific rigor of the program, while think-aloud tests indicated that the mini-program was feasible to deliver and generally acceptable to users. Nevertheless, expert recommendations, usability barriers, and user-derived suggestions highlighted several areas for further refinement.

### Advantages of the Digital Intervention

The “Yi Yang Plan” mini-program was developed through a rigorous, theory- and evidence-based process integrating evidence synthesis, identification of influencing factors, and multidisciplinary collaboration. A prior scoping review identified 4 key elements of PFC [33], which are consistent with the established PFC process model [39], thereby providing a strong conceptual foundation. In addition, a convergent mixed methods study identified key determinants of PFC among frail older adults, and a structural equation model based on the Andersen Behavioral Model of Health Services Use informed the development of stratified intervention pathways. This approach enabled the design of a targeted and differentiated intervention tailored to varying frailty levels. Furthermore, iterative refinement by a multidisciplinary team enhanced both usability and content quality. Collectively, this structured development process offers a replicable framework for designing digital interventions to support PFC.

Expert consultation results indicated high credibility and reliability, reflected by strong engagement and authority coefficients. Experts with substantial clinical and theoretical expertise confirmed the scientific validity of the platform and provided actionable recommendations, supporting the robustness of the development process. The findings from think-aloud testing further demonstrated good feasibility and acceptability. Participants expressed generally positive perceptions and recognized the value of the mini-program, suggesting a willingness among older adults to engage with digital health tools. These findings align with previous studies indicating that older adults perceive supportive digital interventions as useful and acceptable [32,44]. One explanation is that the platform provides accessible information and resources, thereby enhancing the awareness and understanding of PFC [45]. In addition, the involvement of health care professionals in the development process may have strengthened users’ trust, as older adults tend to rely on information from credible and familiar sources [46,47].

### Barriers to Digital Intervention

Despite overall positive findings, several important challenges emerged. First, heterogeneity in PFC readiness influenced user needs. Participants noted that individuals at different stages of PFC require different types of support, which is consistent with prior research [48]. This highlights the importance of tailoring intervention pathways. For individuals with low awareness, interventions should focus on early-stage awareness enhancement, whereas those with higher readiness may benefit more from decision-making and planning support.

Second, disparities in education and digital literacy posed barriers to engagement. Older adults with lower educational attainment may have limited health literacy and digital skills, which can hinder technology adoption [49-51]. Therefore, assessing digital readiness prior to intervention delivery is essential [52]. Complementary support strategies, such as printed manuals, instructional videos, and offline guidance, are necessary to improve accessibility and inclusiveness [53].

Third, although usability received the highest satisfaction ratings and all participants completed the tasks, specific usability issues were identified, including unclear navigation and suboptimal font size. Age-related declines in sensory, motor, and cognitive functions may contribute to these challenges [47,54,55]. Accordingly, future designs should simplify navigation structures, provide clearer operational logic (eg, return and exit functions), and incorporate step-by-step guidance. Established age-friendly design principles, such as larger fonts, high contrast, concise content, and clear labeling, should be consistently applied [56].

Fourth, content quality received relatively lower ratings. While participants acknowledged the comprehensiveness of the information, they reported difficulties in understanding and applying the content. This is likely because the resource module includes policy-level and professionally complex information. Simplifying the language and incorporating patient-friendly expressions may improve comprehension [57]. Additionally, multimedia formats (eg, videos and audio) can enhance engagement and accommodate diverse learning preferences [56]. Furthermore, emerging technologies such as AI could be leveraged to deliver personalized recommendations, improve information filtering, and enhance intervention precision and scalability [58]. Importantly, participants emphasized the need for more practical, actionable resources. Strengthening partnerships with community organizations and service providers is therefore critical to ensure that digital planning can be effectively translated into real-world care arrangements.

Finally, participants highlighted the essential role of adult children in facilitating technology use and care planning. This finding is consistent with previous research showing that older adults rely heavily on family support in PFC [1,44,54]. Integrating adult children as active participants in the intervention may enhance engagement, usability, and overall effectiveness.

### Optimization Strategies

Based on these findings, several optimization strategies are warranted. At the system level, a comprehensive security and privacy protection framework should be implemented, including encryption, role-based access control, data deidentification, and audit logging, to ensure the safe handling of sensitive health information. At the interface level, improvements should focus on enhancing usability and emotional engagement. This includes adopting age-friendly layouts, increasing font size and contrast, simplifying navigation, and incorporating supportive visual and narrative elements to improve the user experience. At the functional level, the platform should further strengthen personalization by integrating periodic assessments, dynamic care plan updates, resource linkage, and historical tracking functions. At the implementation level, additional support tools should be provided to users with limited digital literacy. Moreover, the formal integration of adult children and stronger collaboration with community health care providers are essential to ensure continuity between digital planning and real-world service delivery.

### Limitations

This study has several limitations that should be acknowledged. First, the small sample size limits the generalizability of the findings. As a pilot study, the primary aim was to assess feasibility and acceptability rather than effectiveness. Future research should conduct adequately powered randomized controlled trials to evaluate intervention efficacy. Second, although an iterative design approach was adopted, older adults and their family members were not involved as co-designers during the early development phase. Consequently, some usability issues were only identified during pilot testing. Future studies should incorporate more principles from human factors engineering and adopt participatory design methods to better align the intervention with the needs and capabilities of end users. Third, many participants had prior experience with digital health apps, which may limit the applicability of the findings to populations with lower digital literacy. Future research should intentionally recruit individuals with lower levels of digital skills to ensure that the tool is responsive to the needs of those who require the greatest support.

### Conclusion

Based on a scoping review, analysis of influencing factors, stratified path analysis, as well as a multidisciplinary collaborative development process, this study developed a digital PFC platform for community-dwelling prefrail and frail older adults. It integrates standardized core procedures, stratified precision interventions, and digital platform delivery. The pilot test showed that the “Yi Yang Plan” mini-program demonstrated initial scientific validity, usability, and feasibility. Future versions should focus on improving system architecture, user interface design, platform features, and implementation strategies. The pilot results also provide a solid foundation for further thorough evaluation through randomized controlled trials.

## Supplementary material

10.2196/92492 Multimedia Appendix 1 The interface design on the “Lanhu” platform.

10.2196/92492 Multimedia Appendix 2 “Yi Yang Plan” community management terminal interface.

10.2196/92492 Multimedia Appendix 3 User login and frailty assessment interface.

10.2196/92492 Multimedia Appendix 4 Awareness enhancement interface.

10.2196/92492 Multimedia Appendix 5 Care resources interface.

10.2196/92492 Multimedia Appendix 6 Care decision-making interface.

10.2196/92492 Multimedia Appendix 7 Family care decision checklist.

10.2196/92492 Multimedia Appendix 8 Care plan and updates interface.

10.2196/92492 Multimedia Appendix 9 Care preparation checklist and personal center interface.

10.2196/92492 Multimedia Appendix 10 The methods of the expert authority coefficient.

10.2196/92492 Multimedia Appendix 11 Task list for think-aloud tests.

## Abbreviations

FRAIL: Fatigue, Resistance, Ambulation, Illness, Loss of weight
PFC: preparation for future care
SARC-F: S‌trength, A‌ssistance in walking, R‌ise from a chair, ‌C‌limb stairs, F‌alls
SEM: structural equation model
TAM: technology acceptance model
