# Supplementary material for: Preparation for Future Care in Community-Dwelling Older Adults With Prefrailty and Frailty: Development and Pilot Test of a Digital Intervention
Source: JMIR Aging. 2026 Aug 5;9:e92492. doi: 10.2196/92492 (PMC13440639; doi:10.2196/92492)
Supplement: Supplementary file 1 [file aging-v9-e92492-s007.docx]

Supplementary 2. Family care decision checklist.

| Item | Response options |
| --- | --- |
| 1. With increasing age and disease progression, do you anticipate needing assistance with daily living within the next six months? | ☐ Yes  ☐ No  ☐ Uncertain |
| 2. If you require assistance with daily living within the next six months, who would you prefer to be responsible for providing care? (Multiple responses allowed) | ☐ Adult children  ☐ Spouse  ☐ Friends or acquaintances  ☐ Paid domestic care workers  ☐ Government-provided services  ☐ Short-term caregivers at community day care centers  ☐ Long-term caregivers in residential care facilities  ☐ Other: _________  ☐ Uncertain |
| 3. With increasing age and disease progression, do you anticipate needing improvements to your current living environment within the next six months? | ☐ Yes  ☐ No  ☐ Uncertain |
| 4. If improvements to your living environment are required within the next six months, what would you prefer? (Multiple responses allowed) | ☐ Remain at home with age-friendly home modifications  ☐ Move to live with adult children  ☐ Move to a community-based day care or respite care center  ☐ Move to a senior apartment  ☐ Move to a long-term residential care facility  ☐ Other: _________  ☐ Uncertain |
| 5. With increasing age and disease progression, do you anticipate needing financial management related to care within the next six months? | ☐ Yes  ☐ No  ☐ Uncertain |
| 6. If payment for care services is required within the next six months, what would you prefer as sources of financial support? (Multiple responses allowed) | ☐ Financial support from family members or adult children  ☐ Cash savings or bank deposits  ☐ Public pension insurance  ☐ Commercial pension insurance  ☐ Long-term care insurance  ☐ Investment income (e.g., funds, stocks)  ☐ Purchase or sale of property  ☐ Purchase or transfer of land-use rights  ☐ Re-employment income  ☐ Other: _________  ☐ Uncertain |
| 7. With increasing age and disease progression, do you anticipate needing legal-related services within the next six months? | ☐ Yes  ☐ No  ☐ Uncertain |
| 8. If legal-related services are required within the next six months, what would you prefer? (Multiple responses allowed) | ☐ Development of an advance care plan  ☐ Preparation of a will  ☐ Access to legal aid services  ☐ Other: _________  ☐ Uncertain |
| 9. With increasing age and disease progression, do you anticipate needing health management within the next six months? | ☐ Yes  ☐ No  ☐ Uncertain |
| 10. If health management is required within the next six months, what types of support would you prefer? (Multiple responses allowed) | ☐ Health education  ☐ Medication guidance and management  ☐ Vital sign monitoring  ☐ Development and supervision of exercise programs  ☐ Cognitive training  ☐ Regular follow-up by community physicians  ☐ Nutritional assessment and guidance  ☐ Peer support from individuals with similar conditions  ☐ Other: _________  ☐ Uncertain |
| 11. With increasing age and disease progression, do you anticipate needing psychological counselling within the next six months? | ☐ Yes  ☐ No  ☐ Uncertain |
| 12. If psychological counselling is required within the next six months, what type of support would you prefer? (Multiple responses allowed) | ☐ Professional counselling from a psychologist or psychiatrist  ☐ Non-professional support from social workers or volunteers  ☐ Emotional support from family members  ☐ Other: _________  ☐ Uncertain |
