# Supplementary material for: Preparation for Future Care in Community-Dwelling Older Adults With Prefrailty and Frailty: Development and Pilot Test of a Digital Intervention
Source: JMIR Aging. 2026 Aug 5;9:e92492. doi: 10.2196/92492 (PMC13440639; doi:10.2196/92492)
Supplement: Supplementary file 2 [file aging-v9-e92492-s010.docx]

Supplementary 1. The methods of e**xpert authority coefficient.**
The authority of the experts was evaluated to ensure the reliability of the consultation results. The expert authority coefficient (*Cr*) was calculated using the formula: *Cr* = (*Ca* + *Cs*) / 2, where Ca is the coefficient of the basis for expert judgment, and Cs is the coefficient representing the expert’s familiarity with the research content. Both coefficients were derived from self-assessments by the experts.

The Cs coefficient evaluated experts’ familiarity with elderly care and nursing and geriatric health management, using five levels: very familiar (1.0), familiar (0.8), somewhat familiar (0.6), average familiarity (0.4), and unfamiliar (0.2).

The Ca coefficient assessed the degree to which experts based their judgments on four aspects: theoretical analysis, practical experience, reference to literature, and intuitive judgment. Each aspect was rated according to its influence as large, medium, or small. The scoring criteria are presented in Table 1. A *Cr* value greater than 0.7 indicated high expert authority and reliability of the consultation results.

**Table 1. Scoring criteria for experts’ basis of judgment (***Ca***)**

| **Basis of Judgment** | **Expert Self-Evaluation** | | |
| --- | --- | --- | --- |
|  | Large | Medium | small |
| Practical experience | 0.5 | 0.4 | 0.3 |
| Theoretical knowledge | 0.3 | 0.2 | 0.1 |
| Reference to literature | 0.1 | 0.1 | 0.05 |
| Intuitive judgment | 0.1 | 0.1 | 0.05 |

Note: *Ca* represents the coefficient of the basis for expert judgment. Higher values indicate a greater reliance on practical experience or theoretical knowledge when evaluating the research scheme.
