# Supplementary material for: Preparation for Future Care in Community-Dwelling Older Adults With Prefrailty and Frailty: Development and Pilot Test of a Digital Intervention
Source: JMIR Aging. 2026 Aug 5;9:e92492. doi: 10.2196/92492 (PMC13440639; doi:10.2196/92492)
Supplement: Supplementary file 3 [file aging-v9-e92492-s011.docx]

Supplementary 3. Task list for think-aloud tests.

| No. | Task Name | Task Scenario Description |
| --- | --- | --- |
| **Task 1** | System Familiarization | (a) Search for the mini-program on WeChat, register an account, and log in. (b) Complete and submit personal information. (c) Enable the message subscription function. (d) Open the Yi Yang Plan mini-program. |
| **Task 2** | Awareness Enhancement | (a) Click on the Awareness Enhancement module. (b) Select Preparation for Future Care to review basic knowledge about future care planning.   (c) Select Preparation for Future Care Assessment, complete the assessment, and review the results. |
| **Task 3** | Care Resources | (a) Click on the Care Resources module. (b) Select Daily Care Resources to learn about eldercare service options. (c) Select Residential Care Resources to explore living arrangements for older adults. (d) Select Health Maintenance Resources to review health promotion and preventive care information. (e) Select Financial Security Resources to learn about financial management and available subsidies for older adults. (f) Select Legal Support Resources to understand avenues for safeguarding older adults’ rights. |
| **Task 4** | Care Decision-Making | (a) Click on the Care Decision-Making module. (b) Select Communication Strategies for Care Preparation to review approaches for family discussions about future care. (c) Select Family Care Decision-Making to develop family care preferences. |
| **Task 5** | Care Planning | (a) Click on the Care Planning module. (b) Select Develop a Care Plan to create a personalized care plan for the older adult. (c) Select Review and Update to explore how care plans are revisited and updated. (d) Select Obtain Your Future Care Preparation Report to review the individualized report generated by the system. |
